# Multidisciplinary team diagnosis and treatment of well-differentiated thyroid carcinoma: current landscape and future prospects

**DOI:** 10.1093/oncolo/oyag017

**Published:** 2026-02-06

**Authors:** Yuanyuan Li, Peijie Wang, Jiaxin Cao, Haiyan Liu

**Affiliations:** Department of Nuclear Medicine, First Hospital of Shanxi Medical University, Taiyuan, 030001, China; Shanxi Key Laboratory of Molecular Imaging, Shanxi Medical University, Taiyuan, 030001, China; Department of Nuclear Medicine, First Hospital of Shanxi Medical University, Taiyuan, 030001, China; Shanxi Key Laboratory of Molecular Imaging, Shanxi Medical University, Taiyuan, 030001, China; Department of Nuclear Medicine, First Hospital of Shanxi Medical University, Taiyuan, 030001, China; Collaborative Innovation Center for Molecular Imaging of Precision Medicine, Shanxi Medical University, Taiyuan, 030001, China; Department of Nuclear Medicine, First Hospital of Shanxi Medical University, Taiyuan, 030001, China; Collaborative Innovation Center for Molecular Imaging of Precision Medicine, Shanxi Medical University, Taiyuan, 030001, China; CAEA Center of Excellence on Nuclear Technology Applications for the Diagnosis, Treatment & Transformation of Nuclear Medicine, Taiyuan, 030001, China

**Keywords:** thyroid cancer, multidisciplinary team, oncology, disease management

## Abstract

**Background:**

The incidence of thyroid cancer has increased markedly in recent years, largely driven by well-differentiated thyroid carcinoma (WDTC). WDTC is biologically heterogeneous, with generally favorable prognosis but substantial variability in clinical behavior. Advances in molecular imaging, artificial intelligence-assisted diagnostics, and liquid biopsy have altered diagnostic strategies, while targeted therapy and immunotherapy have expanded treatment options for selected patients with advanced disease. The multidisciplinary team (MDT) model has therefore become an essential component of WDTC management through the integration of expertise from multiple specialties.

**Methods:**

This review examines the role of MDT application in WDTC through analysis of relevant literature and international clinical guidelines, focusing on MDT composition, implementation models, clinical roles across diagnostic and therapeutic pathways, and current limitations. Differences in MDT recommendations among guidelines from various regions were also compared.

**Results:**

MDT involvement supports personalized decision-making in WDTC, particularly in cases with indeterminate diagnosis, risk-adapted treatment selection, recurrent disease, and radioiodine-refractory progression. Persistent challenges include overtreatment of low-risk disease, suboptimal management of high-risk cases, limited MDT implementation in primary hospitals, uneven specialty participation, and variability in decision-making within guideline gray zones.

**Conclusion:**

The MDT model provides a structured framework to improve risk-adapted management of WDTC. Future efforts should prioritize refined risk-stratified MDT models, integration of decision-support tools, and expansion of remote platforms to enhance consistency and quality of management.

Implications for Practice This review highlights how multidisciplinary team (MDT)-based management can improve the diagnosis, treatment, and long-term follow-up of patients with well-differentiated thyroid carcinoma. By integrating expertise from surgery, endocrinology, radiology, pathology, and nuclear medicine, MDT helps reduce both overtreatment in low-risk patients and missed treatment opportunities in high-risk cases. Our comparative analysis of international guidelines also identifies current gaps in real-world MDT implementation and provides practical recommendations to standardize team structure and decision-making. These insights may support clinicians and hospitals in developing more coordinated, patient-centered care pathways that ultimately improve outcomes and quality of life.

## Introduction

According to Global Cancer Statistics 2022, thyroid cancer (TC) is the seventh most common malignancy worldwide, with well-differentiated thyroid carcinoma (WDTC) accounting for the vast majority of cases.^1^ WDTC demonstrates a pronounced female predominance and substantial geographic variability, patterns that largely reflect differences in diagnostic capacity and screening practices.^1^ Clinical outcomes in WDTC are ­generally favorable, with 10-year survival exceeding 99% in microcarcinomas and 85%-98% in localized disease but declining substantially in the presence of distant metastases.^2^ Despite its low disease-specific mortality, WDTC presents significant clinical challenges related to biological heterogeneity, increasing concerns about overtreatment in low-risk disease, and long-term survivorship issues affecting psychological well-being and quality of life.^3^^,^^4^ These complexities underscore the need for multidisciplinary team (MDT) models to enhance diagnostic accuracy, optimize individualized treatment, and standardize follow-up while incorporating psychosocial and lifestyle support.

MDT structures, first introduced in oncology in the 1980s,^5^ are now widely adopted in cancer care. Evidence shows that MDT discussions modify treatment plans in 1.6%-58% of cases^6^ and are associated with improved overall survival (HR 0.67).^7^ Although these models provide valuable reference for complex TC, its indolent yet heterogeneous behavior requires a tailored MDT framework emphasizing molecular profiling, surveillance strategies, and balancing overtreatment with adequate risk control.^8^ Several international guidelines now recommend MDT involvement in TC management, though emphasis varies among regions.^2^^,^^9–12^ As guidelines alone cannot fully address real-world variability, continuous refinement of MDT processes is needed.

## Methods

This study is a narrative review of academic literature published in English over the past decade. Relevant articles were retrieved from PubMed/MEDLINE, focusing on global guidelines for differentiated thyroid carcinoma and the evolving role of MDT. Sources included clinical practice guidelines, consensus statements, original research articles, and expert commentaries. Search strategies combined free-text terms and Medical Subject Headings, including “thyroid cancer,” “thyroid neoplasms,” “guidelines,” “multidisciplinary team,” “diagnosis,” “therapeutics,” “radioiodine therapy,” “follow-up studies,” and “recurrence.” The review was limited to data from peer-reviewed publications and reports presented at scientific congresses, and formal assessment of levels of evidence was not performed.

## Clinical section: core components of WDTC MDT

### Diagnosis: imaging, pathology department, and endocrinology

From a multidisciplinary perspective, imaging techniques for WDTC play a crucial role. Through various imaging techniques, doctors can diagnose, assess, and monitor WDTC more accurately. In clinical practice, thyroid nodules are detected by ultrasound in approximately 50% to 70% of the population, whereas the incidental detection rate of WDTC remains relatively low, ranging from 0.3% to 5%.^13^ Current clinical guidelines recommend against routine screening for WDTC in asymptomatic individuals and emphasize the importance of using risk stratification systems to determine the need for fine-needle aspiration (FNA).^14^

Both the 2015 and 2025 American Thyroid Association (ATA) guidelines emphasize the central role of cervical ultrasound in guiding the diagnosis and management of WDTC.^2^^,^^15^ At the time of initial diagnosis, ultrasound is indispensable for evaluating the primary thyroid lesion, staging cervical lymph nodes, and ultrasound-guided FNA. Based on a systematic evaluation of lesion characteristics, including size, shape, margins, internal echotexture, and evidence of extrathyroidal extension, various countries, and regions have developed ultrasound risk stratification systems to standardize the assessment of malignancy risk and support clinical decision-making. The most widely used systems include the ATA nodular sonographic patterns-based stratification, the American College of Radiology (ACR) Thyroid Imaging Reporting and Data System (TIRADS), the European TIRADS, the Korean TIRADS, and the Chinese TIRADS.^2^^,^^16^^,^^17^ A meta-analysis by Kim et al.,^18^ which included 39 studies, evaluated the diagnostic performance of various ultrasound-based risk stratification systems. The findings indicated that the ACR TIRADS demonstrated the highest sensitivity and specificity for identifying suspicious thyroid nodules. By applying a structured classification approach, the ACR TIRADS was able to reduce the number of unnecessary biopsies by 17.1% to 53.4%, while maintaining a low missed cancer rate of only 2.2%.^19^ However, interobserver variability in ultrasound interpretation continues to be a major challenge, potentially leading to inconsistent clinical management.^20^ This limitation underscores the need for standardized protocols for image acquisition and the strengthening of physician training, with the goal of enhancing diagnostic consistency and reliability.

In response, artificial intelligence (AI), particularly systems based on deep learning algorithms, has emerged as a promising tool to enhance diagnostic consistency, reduce interpretation time, and support more objective decision-making.^21^^,^^22^ Radiomics, which involves the high-throughput extraction of quantitative features from medical images and the integration of machine learning algorithms to develop predictive models, has demonstrated significant potential in the differentiation of benign and malignant thyroid nodules, prediction of pathological subtypes, and assessment of tumor invasiveness.^23^^,^^24^ Looking ahead, continued algorithm optimization and the accumulation of large-scale, high-quality datasets are expected to enhance the added value of AI-assisted diagnostic systems.^25^ Meanwhile, ultrasound technology has evolved from traditional two-dimensional imaging into a multimodal diagnostic platform, incorporating elastography and contrast-enhanced ultrasound.^26^ While each modality offers incremental benefits in the evaluation of thyroid nodules, none can fully replace conventional grayscale ultrasound, which remains the cornerstone of initial assessment.^27–29^ Further large-scale, prospective studies are needed to clarify the indications, limitations, and diagnostic performance of these emerging technologies.^30^ How to better interpret and develop a model that diagnoses and follow up, including ultrasound, requires the joint exploration of data from multiple departments.

Computed tomography (CT) and magnetic resonance imaging (MRI) serve as supplementary modalities to ultrasonography in the assessment of thyroid nodules. A meta-analysis encompassing 31 942 patients demonstrated superior sensitivity of CT (61%) compared to ultrasound (41%) for detecting central lymph node metastasis.^31^ MRI exhibited significantly higher sensitivity than ultrasound for identifying gross extrathyroidal extension (85.4% vs. 66.7%, respectively).^32^ However, neither contrast-enhanced CT nor MRI surpasses ultrasound in differentiating benign from malignant nodules. For patients with evidence of more advanced locoregional disease, selective use of CT or MRI may be considered to assist in preoperative planning.^2^

When suspicious thyroid nodules are identified via ultrasound examination, FNA biopsy is routinely employed as the subsequent diagnostic procedure. FNA results are standardized using established reporting frameworks, with the Bethesda System for Reporting Thyroid Cytopathology (TBSRTC), a 6-tier classification, being the most widely adopted. However, this system exhibits significant heterogeneity in malignancy risk stratification, particularly for categories III (Atypia of Undetermined Significance/Follicular Lesion of Undetermined Significance) and IV (Follicular Neoplasm/Suspicious for Follicular Neoplasm).^33^ To enhance preoperative diagnostic accuracy, complementary strategies may be integrated, including but not limited to adjunctive ultrasound techniques (eg, elastography), immunohistochemical analysis, molecular testing, Raman spectroscopy, and core needle biopsy.^34–36^ Repeat FNA may also be considered when initial results are inconclusive. Over recent years, multiple research teams have validated diverse molecular diagnostic models—including MPTXv1, MPTXv2, ThyroSeq v3, Afirma, and ThyroSPEC—demonstrating their capacity to enhance the diagnostic accuracy of FNA cytology to varying degrees (the positive predictive value: 76%-99%).^37–40^ Furthermore, AI algorithms integrating ultrasonographic features with clinical parameters show potential to reduce unnecessary diagnostic surgeries.^41^^,^^42^ The synergistic integration of these technologies is fundamentally reshaping the diagnostic paradigm for thyroid nodules while concurrently enabling prediction of tumor biological behavior and evidence-based therapeutic guidance. Future identification of novel molecular markers promises to further refine the molecular taxonomy of WDTC.

Thyroid hormones and related antibodies are more commonly utilized for detecting postoperative recurrence but are not reliable for distinguishing benign from malignant conditions preoperatively. Elevated thyroglobulin (Tg) levels in FNA washout fluid (FNA-Tg) can indicate lymph node metastasis; however, there is currently no standardized clinical cut-off value, and large-scale studies are still needed for validation. The investigation of blood biomarkers offers a non-invasive and repeatable approach for the diagnosis and monitoring of TC, including circulating tumor DNA (ctDNA), circulating tumor cells (CTCs), circulating mitochondrial DNA, exosomes, and microRNAs (Table 1 lists some important references).^43–48^ These techniques are particularly valuable for patients in whose tissue acquisition is challenging or for those requiring long-term surveillance, thus addressing critical limitations in clinical practice. These breakthroughs in the diagnosis of TC have not only addressed many long-standing challenges in clinical practice but have also established a foundation for individualized treatment in the era of precision medicine. In the future, multimodal data fusion, the establishment of standardized processes, and large-scale clinical validation will be the main directions of development in this field.

**Table 1.** Main contents of articles related to blood markers.

Publication time	Indicator	Population	Clinical application	The best cut-off value	Reference
**2023.11**	FNA-Tg	WDTC patients with suspicious lymph nodes (*N* = 1106)	FNA-Tg was indicative of cervical lymph nodes metastasis in WDTC patients, and this efficacy was not affected by FNA-antithyroglobulin antibody.	25.17 µg/L	Liu et al.^46^
**2021.07**	cell-free DNA integrity index (cfDI)	Patients with different types of thyroid nodules (*N* = 85)	cfDI、cell-free DNA Alu244 and cell-free DNA Alu83 were associated with tumor size and capsular invasion.	Cell-free DNA Alu244 ≥ 0.95 ng/ml and cfDI ≥ 0.3 cfDI ≥ 0.3 can distinguish TC from follicular neoplasm/suspicious for follicular neoplasm.	Higazi et al.^47^
**2023.10**	CTCs、ctDNA、cfDI	Meta-analysis of 36 literatures (*N* = 2566)	Higher levels of CTCs and cfDI and ctDNA with mutations have beneficial value for the management of TC.	–	Zeyghami et al.^45^
**2024.01**	circulating small extracellular vesicle miRNA (CirsEV-miR)	Patients with thyroid nodules who planned to undergo thyroidectomy were pathologically confirmed to have papillary thyroid carcinoma (FTC) or follicular thyroid adenoma after the operation (*N* = 191)	The CirsEV-miR classified combined with ultrasonography is helpful for differentiating FTC from follicular thyroid adenoma.	This classifier contains miR-127-3p, miR-223-5p, miR-432-5p, miR-146a-5p and miR-151a-3p.	Li et al.^44^
**2024.09**	miR-146a-5p and miR-221-3p	Patients with papillary thyroid carcinoma (PTC) who planned to undergo thyroidectomy (*N* = 117)	Both may be valuable biomarkers for the diagnosis and follow-up of PTC patients.	miR-146a-5p ≥ 768,545copies/mL miR-221-3p ≥ 389,331copies/mL	Verrienti et al.^43^
**2019.07**	leukocyte mitochondrial DNA copy number	Patients with TC (*N* = 808)	The increase in leukocyte mitochondrial DNA copy number was an independent risk factor for TC.	–	Zheng et al.^48^

Abbreviations: CTCs, circulating tumor cells; ctDNA, circulating tumor DNA; FNA-Tg, fine needle aspiration washout fluid; TC, thyroid cancer; WDTC, well-differentiated thyroid carcinoma.

### Treatment: surgery, endocrinology, the department of nuclear medicine, oncology, and the interventional radiology department

With the advent of the era of precision medicine, the management of WDTC is progressively transitioning toward an MDT-based decision-making model. Treatment strategies have evolved from traditional surgery and radioactive iodine therapy to a multi-modal comprehensive approach encompassing targeted therapy, immunotherapy, and gene therapy, enabling the formulation of individualized treatment plans.^2^^,^^8^ Initial management options now include active surveillance (AS), immediate surgery, and conversion surgery. A 30-year follow-up study demonstrated no statistically significant difference in clinical oncologic outcomes between low-risk WDTC patients who underwent AS and those who received immediate surgery.^49^ However, patients’ anxiety and fear regarding the disease and its treatment are critical factors influencing the acceptance of AS.^50^ Therefore, clinical decision-making should be grounded in thorough patient education and incorporate assessments of patient compliance and psychological readiness.

With advances in thermal ablation technologies, radiofrequency ablation (RFA) and microwave ablation have been increasingly applied in patients with papillary microcarcinomas of the thyroid, particularly in those experiencing anxiety or local tumor progression during AS.^51^^,^^52^ A meta-analysis evaluating RFA demonstrated a complete tumor disappearance rate of 79% with RFA, compared with 100% for surgery and 0% for AS. Tumor progression rates were 1.5%, 3%, and 5%-10%, respectively, while overall complication rates were 2.7%, 30% (primarily hypothyroidism), and 0%.^52^ Furthermore, a propensity score-matched study involving 844 patients showed that RFA was associated with significantly shorter hospital stays (0 vs. 7 days) and lower treatment costs ($2,035.7 vs. $2,269.1) compared with surgery.^53^ Collectively, current evidence suggests that in patients with papillary microcarcinomas of the thyroid, RFA offers distinct advantages over surgery, including fewer complications, superior functional preservation, faster recovery, and lower costs. In comparison with AS, RFA provides immediate tumor eradication, thereby alleviating psychological burden. However, patients undergoing RFA require long-term follow-up to clarify the risks of lymph node metastasis, local tumor progression, and to further evaluate long-term cost-effectiveness.

While the indications and extent of traditional surgical procedures have been clearly defined by various clinical guidelines, the issue of overtreatment remains a significant concern in clinical practice.^2^ Multiple retrospective cohort studies revealed that in appropriately selected patients, there was no statistically significant difference in recurrence-free survival between lobectomy and total thyroidectomy (TT), and it was not affected by age or tumor size.^54^^,^^55^ However, notable differences exist in the incidence of postoperative complications between the two surgical approaches. TT carries a higher risk of adverse outcomes, including recurrent laryngeal nerve injury, hypothyroidism, and hypoparathyroidism. From a health economics perspective, utility values (ranging from 0 to 1) are employed as quantitative indicators to measure patient preference and quality of life associated with specific health states. Evidence indicates that patients undergoing lobectomies achieve a utility value of approximately 0.99, nearly equivalent to complete health. By contrast, complications following TT can markedly reduce utility values: unilateral recurrent laryngeal nerve injury is associated with a value of 0.627, bilateral injury as low as 0.205, and hypoparathyroidism 0.778.^56^ These findings underscore the profound negative impact of surgery-related complications on patients’ long-term quality of life. Therefore, in clinical decision-making, individualized risk assessment should guide the careful selection of surgical strategy, balancing oncologic outcomes with health-related quality of life (HRQoL) considerations.^57^

In parallel, innovative surgical techniques are reshaping conventional thyroid surgery. Approaches, such as transaxillary, suprasternal fossa, thoracic, breast, chest-breast, transoral endoscopic, or robotic-assisted thyroidectomy have emerged as safe and feasible alternatives to open TT, effectively reducing the physical and psychological burden associated with traditional open surgery.^58–62^ In conclusion, the diagnosis and treatment of WDTC are undergoing a paradigm shift from a disease-centered model to a patient-centered approach.^63^ Moving forward, personalized treatment strategies should be guided by precise preoperative assessments, shared decision-making with patients, and close multidisciplinary collaboration, ultimately aiming to extend survival and enhance quality of life.

Radioactive iodine (RAI) therapy remains a cornerstone in the postoperative management of patients with WDTC. However, its application is increasingly moving toward personalization and selectivity. During the thyroid-stimulating hormone (TSH) stimulation phase preceding RAI, multiple studies have demonstrated that the ablation success rate and disease control rate are comparable between recombinant human TSH (rhTSH) and thyroxine withdrawal.^64^ Notably, rhTSH has been shown to reduce hospitalization duration and minimize adverse effects.^65^ Nevertheless, its widespread clinical adoption is limited by healthcare system, cost-effective considerations and insufficient evidence in high-risk populations. According to the ATA guideline recurrence risk stratification, RAI therapy is strongly recommended for high-risk patients to improve long-term outcomes. In contrast, for low-risk WDTC patients who have undergone complete thyroidectomy, current evidence suggests no statistically significant difference in 5-year recurrence rates between the non-ablation group (97.9%) and the ablation group (96.3%).^66^ Similarly, event-free survival—defined by structural or biochemical endpoints—also shows no significant difference (93.2% vs. 94.8%).^67^ These findings support the safe omission of postoperative RAI in low-risk patients, thereby reducing hospitalizations and treatment-associated morbidity. Although some studies have included intermediate-risk patients and reported similar outcomes, their conclusions are limited by short follow-up durations and small sample sizes, which restrict broader clinical applicability.^68^ Meta-analyses evaluating RAI activity in low- to intermediate-risk patients have revealed no significant difference in long-term cure rates between low-dose RAI (≤3 GBq) and high-dose RAI (>3 GBq) (relative risk 0.88, *P *= .50).^69^ These results underscore the importance of accurate risk stratification to identify patients who may benefit from RAI and to guide appropriate dosing strategies. Recent studies suggest that advanced models incorporating peri-RAI findings—such as pre-RAI Tg levels ≥10 ng/mL or post-therapeutic^131^ whole-body scan showing extrathyroidal uptake-can enhance prognostic accuracy, leading to reclassification of 27% of low-risk and 46% of intermediate-risk patients into higher-risk categories. However, these models did not incorporate single photon emission computed tomography (SPECT)/CT imaging, potentially underestimating the detection of micro metastases.^70^ Future efforts should focus on integrating a broader range of parameters-including postoperative histopathology, stimulated Tg levels, and molecular profiles-to enable dynamic and individualized risk stratification. This approach will facilitate the identification of high-risk patients who may benefit from intensified therapy while avoiding overtreatment in low- and intermediate-risk populations.

Over the past few decades, substantial progress has been achieved in the management of advanced differentiated TC, fundamentally transforming the traditional treatment paradigm. The US Food and Drug Administration and the European Medicines Agency have approved multiple targeted agents for clinical use (Table 2 summarizes key references).^71–73^ These include first-line multi-target tyrosine kinase inhibitors, such as sorafenib and Lenvatinib, and the second-line agent cabozantinib.^74–76^ For patients harboring specific genetic alterations, such as RET fusion-positive disease, highly selective RET inhibitors, including selpercatinib and pralsetinib, have also received regulatory approval.^77^ Redifferentiation therapy—such as the combination of dabrafenib and trametinib—can restore the iodine-uptake capacity of tumors, allowing 33-95% of RAI-refractory patients to regain eligibility for RAI therapy.^78^^,^^79^ This approach is particularly effective in patients with BRAF^V600E^ mutations. Immunotherapies, including PD-1/PD-L1 and CTLA-4 inhibitors, as well as novel cellular approaches, have also demonstrated potential in refractory TC.^80^ Current research priorities focus on optimizing treatment timing, implementing precision medicine guided by molecular profiling, developing multimodal combination strategies, and elucidating mechanisms of drug resistance and biomarker development. While these advances have markedly improved survival outcomes and quality of life, challenges remain, including acquired resistance, treatment-related toxicities, and health-economic considerations.^81^ Future clinical trials should aim to refine treatment sequencing and combination regimens while also exploring novel mechanisms—such as epigenetic modulation—to further enhance therapeutic efficacy.

**Table 2.** Therapeutic targets and drugs corresponding to different mutation types.

Mutational hallmarks	Pathway	Target	Efficacy
**–**	Vascular endothelial growth factor (VEGF) pathway	VEGF, FGFR, PDGFR (multi-targeted tyrosine kinase inhibitors, MKI, lenvatinib, sorafenib. cabozantinib, etc.)	Lenvatinib: objective response rate (ORR) 64.8%-69.9%^82^ Sorafenib: ORR 24%^83^ Cabozantinib ORR 9%-15%^84^
**BRAF^v600e^**	Mitogen-activated protein kinase (MAPK) pathway	BRAF inhibitors (dabrafenib), MEK inhibitors (trametinib)	Dabrafenib: ORR 42% Dabrafenib + trametinib: ORR 48%^78^
**RAS fusion**	MAPK and PI3K-AKT pathways	MEK inhibitors (trametinib), RAF inhibition (vemurafenib and dabrafenib)	Relatively high RAI uptake recovery, especially for RAS-like tumors^85^
**RET fusion**	RTK-MAPK pathway	RET inhibitors (selpercatinib and pralsetinib)	Selpercatinib: ORR 79%-84.5%^77^^,^^86^ Pralsetinib: ORR 89%^87^
**NTRK fusion**	MAPK and PI3K-AKT pathways	NTRK inhibitors (larotrectinib and entrectinib)	Larotrectinib: ORR 86%^88^
**PAX8-PPARγ fusion**	PPARγ pathways	PPARγ agonists (pioglitazone, experimental)^89^	–
**TP53 mutation**	Cell cycle and DNA repair disorders	–	Associated with poor prognosis
**TERT promoter mutations**	Reactivate TERT transcription	–	Associated with aggressive disease and poor radioiodine response
**PI3K-AKT mutation**	PI3K-AKT-MTOR pathway	AKT/MTOR inhibitors (everolimus, temsirolimus, sirolimus, experimental)^90^	–
**SWI-SNF gene mutations**	Chromatin and transcriptional changes	-(experimental)	Redifferentiation therapy was ineffective^91^

### Dynamic surveillance: serology and imaging

In the dynamic surveillance of WDTC, serological and imaging modalities remain central to postoperative monitoring. For patients undergoing TT with RAI, Tg and Tg antibodies (TgAb) serve as the primary biomarkers. Criteria for no evidence of disease generally include non-stimulated Tg <1 ng/mL or stimulated Tg <0.4-2 ng/mL, whereas higher levels—such as non-stimulated Tg >2 ng/mL or stimulated Tg >2-10 ng/mL—suggest possible recurrence. TgAb >60 ng/mL may both interfere with Tg assays and indicate persistent disease activity.^92^ Ultrasonography is the first-line modality for detecting locoregional recurrence,^93^ while diagnostic RAI whole-body scans, CT/MRI, and positron emission tomography-CT are reserved for evaluating distant metastases,^94–97^ especially when biochemical recurrence occurs despite negative ultrasound findings. Risk-stratified surveillance typically recommends comprehensive assessment at 6-12 months after surgery for high-risk patients, followed by evaluations every 6-12 months whereas low-risk patients may undergo annual follow-up.^92^

Monitoring becomes more complex in patients treated with hemithyroidectomy or TT without RAI. In these settings, cytopathology and structural imaging play a greater role,^66^ as residual thyroid tissue limits the interpretability of Tg levels, although a doubling of baseline Tg may serve as a warning signal.^98^ Surveillance in such patients emphasizes longitudinal comparison against preoperative baselines rather than strict definitions of persistence versus recurrence.^99^

Management of TSH also follows risk stratification: high-risk patients require suppression to <0.1mIU/L, whereas intermediate- or low-risk patients may be maintained within 0.5-4.0mIU/L, with no significant recurrence difference between 0.5-2.0mIU/L and 2.0-4.0mIU/L.^100^ TSH >4.0mIU/L should be avoided due to increased recurrence risk (Table 3 summarizes the serological index thresholds under different treatment regimens and risk stratification states). Despite these principles, inconsistencies persist among studies and guidelines regarding Tg thresholds, surveillance intervals, and risk criteria.^92^^,^^98^ As the number of patients not receiving RAI increases, establishing tailored monitoring standards is increasingly important. Future priorities include quantifying residual thyroid tissue, identifying reliable serological warning thresholds, optimizing imaging strategies, and developing international consensus to harmonize monitoring practices and improve recurrence detection.

**Table 3.** The serological index thresholds under different treatment regimens and risk stratification states.

Population	Risk stratification	Stage	No-stimulated Tg	Stimulated Tg	Additional testing^92^ ^,^ ^98^	TSH	TSH
						A previous recommendation^15^ ^,^ ^100^	An updated recommendation^100^
**Active surveillance or ablation**	–	–	–	–	Ultrasound	–	–
**Lobectomy**	–	–	–	–	Ultrasound	0.5-2 mIU/L	0.5-4 mIU/L
**Follow-up should primarily rely on imaging examinations; Tg serves only as a reference (a doubling from baseline suggests possible recurrence)**							
**TT without RAI**		No evidence of disease	<0.5-1.0 ng/mL	<1.0-2.0 ng/mL	Ultrasound + no-stimulated Tg (every 6-12 months post-surgery) + Stimulated Tg (every 1-2 years post-surgery)	0.5-2 mIU/L	0.5-4 mIU/L
		Suspected recurrence	>1.0 ng/mL	>2.0 ng/mL	Imaging		
**Follow-up should primarily rely on imaging examinations; Tg serves only as a reference (a doubling from baseline suggests possible recurrence)**							
**TT+pre-RAI**	–	–	–	>1-2.5 ng/mL (high risk)	^131^I whole-body scan	>30 mIU/L	>30 mIU/L
**TT+RAI**	Low-risk	No evidence of disease	<0.1-1.0 ng/mL	<0.4-2.0 ng/mL	Ultrasound (annually)	0.5-2 mIU/L	0.5-4 mIU/L
		Suspected recurrence	>1.0-10.0 ng/mL	>2.0-10.0 ng/mL	Ultrasound+SPECT/CT/MRI		
**TT+RAI**	Intermediate-risk	No evidence of disease	<0.2-1.0 ng/mL	<0.4-2.0 ng/mL	Ultrasound (every 6-12 months post-surgery) + stimulated Tg (every 1-2 years post-surgery)	0.5-2 mIU/L	0.5-4 mIU/L
		Suspected recurrence	>1.0 ng/mL	>2.0 ng/mL	Imaging or cytology/pathology		
**TT+RAI**	High-risk	No evidence of disease	<0.1 ng/mL	<0.1-0.5 ng/mL	Ultrasound + no-stimulated Tg (every 6-12 months post-surgery) + Stimulated Tg (every 1-2 years post-surgery)	<0.1 mIU/L	<0.1 mIU/L
		Suspected recurrence	>0.1-1 ng/mL	>1.0-2.0 ng/mL	Imaging or cytology/pathology	<0.1 mIU/L	<0.1 mIU/L

Abbreviations: CT, computed tomography; MRI, magnetic resonance imaging; RAI, radioactive iodine; SPECT, single photon emission computed tomography; Tg, thyroglobulin; TSH, thyroid-stimulating hormone; TT, total thyroidectomy.

### Others: psychology, sleep, nutrition, rehabilitation, traditional Chinese medicine, etc

With the rising incidence of WDTC and prolonged patient survival, follow-up systems that address HRQoL must integrate assessments across physiological, psychological, and social dimensions. Clinical evaluations should prioritize patient-reported outcome measures, encompassing economic stress, anxiety, depression, fear of recurrence, and functional impairments, such as voice changes, dysphagia, scarring, fatigue, weight gain, and insomnia associated with diagnosis and treatment.^3^^,^^101^ Evidence indicates that significant declines in health utility values occur during six months pre-/post-surgery (−0.023-0.222 vs. 0.856 in healthy individuals), during peri-RAI (0.185-0.222), and due to chemotherapy-related toxicities (0.042-0.177).^56^ Fear of recurrence is especially prevalent among younger patients, women, those with lower educational attainment, recent diagnoses, and individuals approaching follow-up visits, underscoring the need for early psychological intervention and enhanced physician-patient communication.^102^ Financial toxicity is most pronounced among younger patients, those without insurance, rural residents, individuals undergoing long-term TSH suppression, and those subjected to frequent imaging follow-ups.^103^ Multidisciplinary strategies are urgently needed to implement cost transparency, insurance navigation, remote follow-up services, and integrated psycho-economic interventions.^103–105^ Preliminary evidence suggests that mobile health interventions promoting behavioral change can significantly improve anxiety, depression, and overall HRQoL.^106^ Future research should focus on incorporating patient-reported outcome measures into individualized treatment plans and conducting multicenter trials to validate the effectiveness of digital health intervention models.

Lifestyle factors play a pivotal role in the recovery process.^107^ Sleep disturbances, often linked to thyroid dysfunction or treatment side effects, may be alleviated by optimizing thyroid hormone levels and managing stress.^108^ Regular physical activity contributes to improved physical fitness, emotional regulation, and bone health.^109^^,^^110^ Nutritionally, a balanced diet remains fundamental, with particular emphasis on adequate calcium and vitamin D supplementation and weight control.^111–113^ The gut microbiota has emerged as a novel area of interest, with studies indicating potential associations between microbiome composition, TC, and intestinal dysbiosis.^114^^,^^115^ Treatments such as surgery, RAI, and immunotherapy may further alter microbial communities; however, clinical translational evidence remains limited and warrants further investigation.^116^^,^^117^ Adjunctive therapy with traditional Chinese medicine can be considered within the framework of evidence-based Western medical care, particularly for mitigating treatment-related side effects.^118^^,^^119^

In summary, optimal recovery from TC requires a comprehensive, multidisciplinary approach integrating standardized medical care, psychological support, healthy lifestyle practices, and reliable health information channels. However, current research predominantly focuses on individual lifestyle interventions rather than conducting comprehensive evaluations, and outcomes are often influenced by multiple confounding factors. Future studies should incorporate more objective quantitative indicators to assess the lifestyles and should employ robust randomized controlled trials to strengthen the relatively limited evidence base regarding holistic lifestyle management.

## The value and challenges of MDT in WDTC

**Figure 1.**
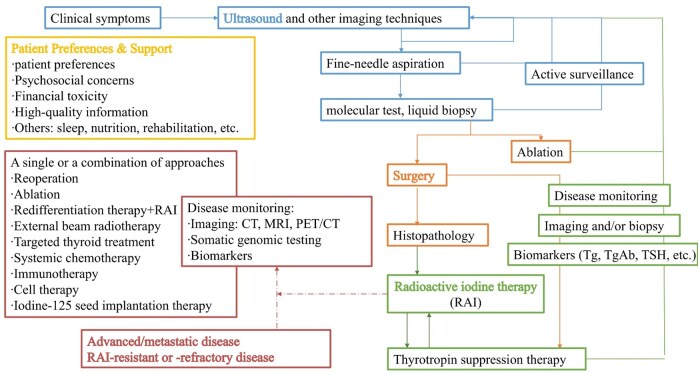
Thyroid cancer multidisciplinary team framework. CT, computed tomography; MRI, magnetic resonance imaging; PET, positron emission tomography; Tg, thyroglobulin; TgAb, Tg antibodies; TSH, thyroid-stimulating hormone.

An MDT is composed of experts from multiple fixed specialties who meet regularly to formulate evidence-based treatment strategies that are implemented independently or collaboratively. In TC, MDT generally include specialists in endocrinology, ­surgery, pathology, radiology, nuclear medicine, medical oncology, and radiation oncology.^15^ By integrating molecular testing, imaging assessment, surgical decision-making, targeted therapy, and long-term follow-up, MDT provide individualized management across risk categories and overcome the limitations of single-specialty perspectives^120^ (Figure 1 shows the framework to offer precise management to TC patients).

Patient-reported data indicate that the MDT model is widely supported, with 89.3% of TC patients expressing willingness to participate in treatment decisions and valuing emotional support (66.6%) and clear communication (82.7%).^121^ Management changes occur in approximately 15% of cases after MDT discussion, most commonly involving additional imaging or RAI treatment adjustments.^122^ Physicians also report improvements in testing strategies, imaging optimization, and adherence to guideline-based protocols.^123^

However, several challenges persist. MDT discussions may insufficiently address recurrence risk or preoperative planning, and participation from key specialties may be inconsistent. Resource disparities limit implementation in primary ­hospitals,^124^ and in guideline “gray zones,”^125^ MDT decisions may be overly conservative, potentially contributing to overtreatment. Digital tools, such as clinical decision support systems and virtual MDT platforms have shown promise in enhancing efficiency, reducing bias, and improving guideline compliance.^126–128^

International guidelines uniformly acknowledge the value of MDTs but differ in emphasis: The National Comprehensive Cancer Network guideline mandates MDT involvement for advanced or high-risk cases^129^; Chinese guidelines encourage broader implementation, including primary care settings^130^; Japanese guidelines focus on simplified microcarcinoma management^17^; and European guidelines highlight high-volume centers and molecular profiling integration.^120^ Collectively, these guidelines converge on the importance of MDT but differ in operational requirements, clinical scope, and integration of supportive care (Table 4 provides a comparative summary of MDT applications in TC guidelines from different countries and regions over the past decade).

**Table 4.** Clinical scenarios involving MDT in some guidelines over the past decade are listed.

Guidelines	Emphasize the activation of MDT in the following circumstances
**American Thyroid Association (2025)^2^**	Surgical decision-making, with an emphasis on comprehensive preoperative discussions and the incorporation of patient preferences to ensure individualized and evidence-based overall treatment strategy and follow-up plan Development of follow-up strategies, including the design of surveillance protocols and the determination of appropriate timing and indications for re-intervention Personalized management of patients with unresectable and/or metastatic TC, where multidisciplinary expertise is essential to optimize therapeutic sequencing, integrate systemic therapies, and improve long-term outcomes
**National Institute for Health and Care Excellence (2022)^9^**	The provision of high-quality information and support to patients with TC The management of treatment and follow-up for TC patients, particularly those at the highest risk of recurrence or with persistent biochemical or structural disease
**National Comprehensive Cancer Network (2025)^129^ and American Head and Neck Society Endocrine Surgery Section and International Thyroid Oncology Group (2022)^10^**	The management of patients with advanced/metastatic disease, with particular emphasis on molecular monitoring and systemic therapeutic interventions
**Internation (2021)^131^**	The selection of surgical approaches The management of postprocedural expectations regarding clinical outcomes, radiographic findings, and biochemical indicators
**Internation (2019)^132^**	The accurate assessment of post-operative disease status The selection of administered activity for adjuvant therapy
**Internation (2020)^11^**	Individualized risk-stratified management prior to treatment initiation
**European Society of Endocrine Surgeons (2024)^120^**	The management of advanced TC, including the performance of diagnostic and molecular testing, the formulation of treatment strategies, and the planning of follow-up and adjuvant therapy
**The Japan Association of Endocrine Surgery (2024)^17^**	The selection of treatment options for advanced differentiated TC
**European Organization for Research and Treatment of Cancer (2019)^12^**	The determination of follow-up tools and schedules for patients with TC The management of patients with advanced/metastatic disease
**The Japan Association of Endocrine Surgery (2021),^133^ European Organization for Research and Treatment of Cancer (2023),^134^ and Korean Thyroid Association (2025)^135^**	The active surveillance for adult patients with low-risk papillary thyroid microcarcinoma Emphasis on the selection of treatment options for adult patients with low-risk papillary thyroid microcarcinoma
**Canada (2024)^136^**	The formulation of diagnostic protocols, therapeutic regimens, and logistical/implementation plans The selection of treatment options for RAI-resistant or -refractory differentiated thyroid carcinoma
**Europe (2025)^137^**	The making of testing and treatment decisions throughout the entire clinical journey The performance of appropriate and timely biomarker testing and molecular detection The early identification of TC patients at risk of developing RAI-refractory disease, as well as the development of practical referral and implementation strategies
**Internation (2025)^138^ and Korea (2025)^16^**	The selection of radiofrequency ablation as a therapeutic option for eligible TC patients
**China (2022^130^, 2025^139^)**	The selection of treatment options for TC patients with persistent, recurrent, or metastatic disease The planning and implementation of postoperative treatment and follow-up for all subtypes of TC

Abbreviations: MDT, multidisciplinary team; RAI, radioactive iodine; TC, thyroid cancer.

Future MDT development should emphasize standardized structures, clearly defined roles, routine meetings, and unified decision-making pathways. Incorporating AI-assisted systems and telemedicine can expand access and consistency, while integrating supportive specialties—such as psychology, nutrition, and rehabilitation—will facilitate a shift toward a fully patient-centered model.

## Conclusion

The management of WDTC is entering a new era defined by precision medicine and multidisciplinary collaboration. MDT provides a framework for individualized, evidence-based decision-making, improving diagnostic accuracy, therapeutic efficacy, and patient satisfaction. Nevertheless, their implementation is limited by subjective decision-making in ambiguous clinical scenarios, unequal resource distribution, and the absence of standardized operational models.

Future efforts should focus on promoting both the standardization and humanization of MDT practice. On one hand, technological innovations—including AI, clinical decision support systems, and virtual consultation platforms—should be leveraged to improve decision-making consistency and efficiency. On the other hand, structural optimization, international collaboration, and the incorporation of supportive specialties are essential for achieving comprehensive, equitable, and patient-centered care.

Ultimately, the evolution of MDT practice must move beyond a disease-centered framework toward a truly patient-centered paradigm, integrating clinical evidence with patient preferences. Such an approach will help overcome existing limitations, reduce regional disparities, and ensure sustainable improvements in the quality and equity of TC management.

## Data Availability

No data were generated for this article; published references are cited at the end of the article.
